# Supplementary figures and images for: Loss of Function of the Neural Cell Adhesion Molecule NrCAM Regulates Differentiation, Proliferation and Neurogenesis in Early Postnatal Hypothalamic Tanycytes
Source: Front Neurosci. 2022 Apr 7;16:832961. doi: 10.3389/fnins.2022.832961 (PMC9022636; doi:10.3389/fnins.2022.832961)

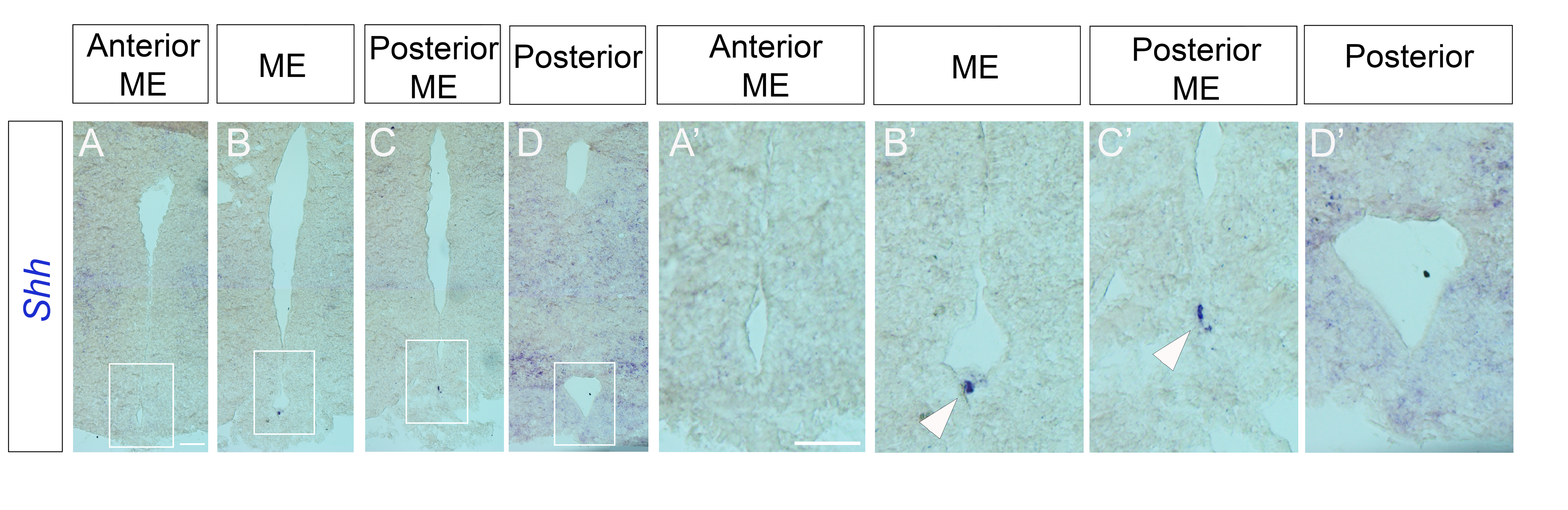

Supplement: Supplementary Figure 1 — Medial-most cells in the tuberal hypothalamus express Shh. (A–D′) Representative examples from consecutive serial adjacent coronal sections (same sample as shown in Figures 1M–T′) across the AME, ME, PME and posterior tanycyte-rich hypothalamus, analyzed for expression of Shh. Boxed regions in panels (A–D) shown at high power in panels (A′–D′). Arrowheads in panels (B′,C′) point to Shh-expressing VZ cells (n = 3 mice; images from a single mouse). Scale bar: 100 μm. [file Image_1.jpeg]

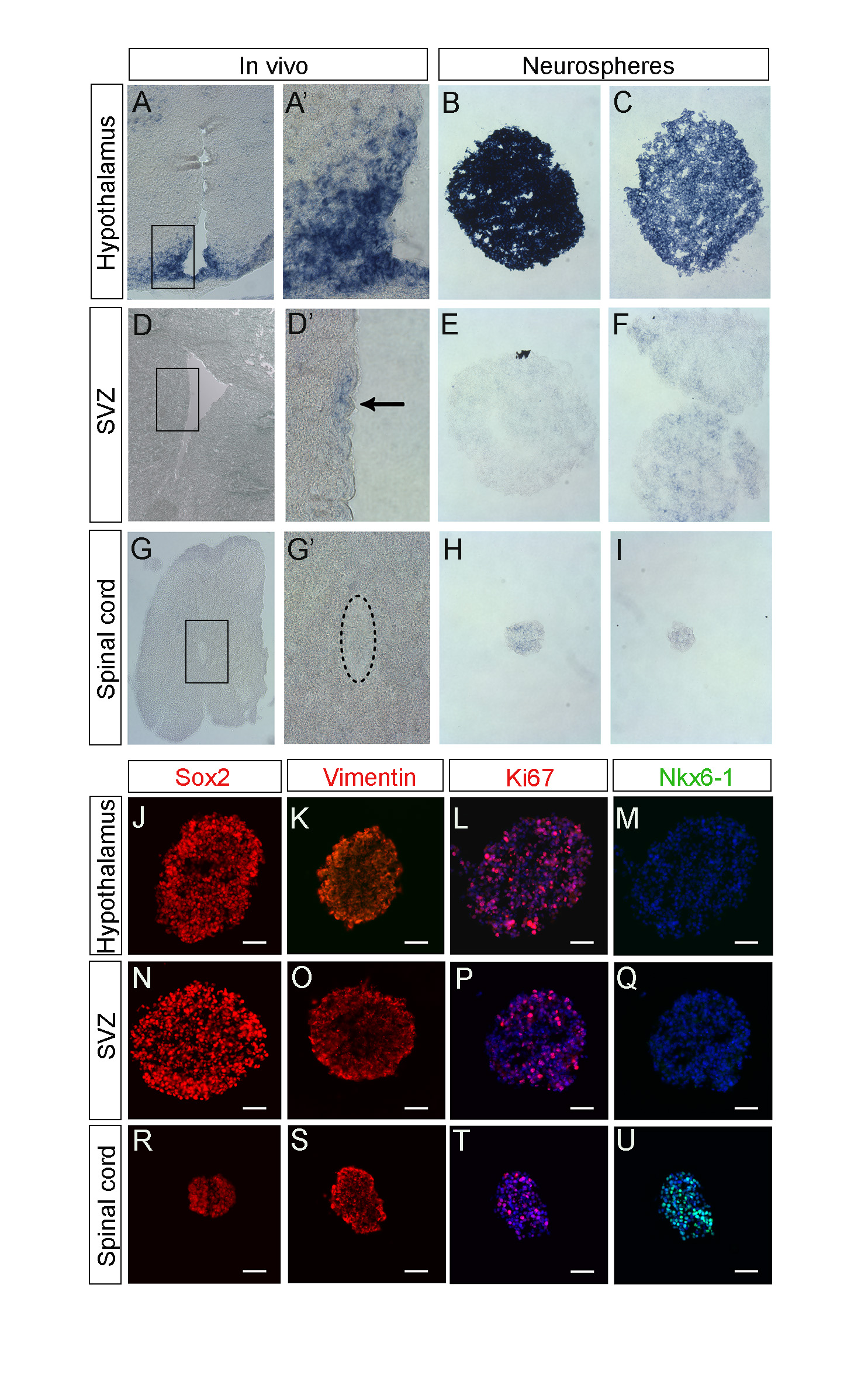

Supplement: Supplementary Figure 2 — Selective expression of regional markers in neurospheres cultured from different regions of the CNS. (A,D,G) Coronal/transverse sections through 8–10 week adult tuberal hypothalamus (A), lateral ventricle of the brain (D) and spinal cord (G), after in situ hybridization to detect Six3. Boxed regions shown in high power in panels (A′,D′,G′). Arrow in panel (D′) points to weak expression of Six3 in the subventricular zone (SVZ). Dotted outline in panel (G′) shows ependymal region (n = 4 mice). (B,C,E,F,H,I) Representative examples of sections through neurospheres cultured under non-differentiated conditions, analyzed by in situ hybridization for Six3 expression (2 from each region to show range; n = 10 neurospheres from each of 3 replicates). Only hypothalamic neurospheres show strong Six3 expression. (J–U) Representative examples of sections from neurospheres from hypothalamus, SVZ of the lateral ventricle, and spinal cord ependymal region, cultured under non-differentiated conditions, analyzed by immunohistochemistry. Neurospheres from all regions express the pan-neural stem cell markers, Sox2 and Vimentin, and proliferate (Ki67-positive), but only those from spinal regions express Nkx6-1 (n = 5 neurospheres analyzed from each of 3 replicates; 30 fields of view total). [file Image_2.jpeg]

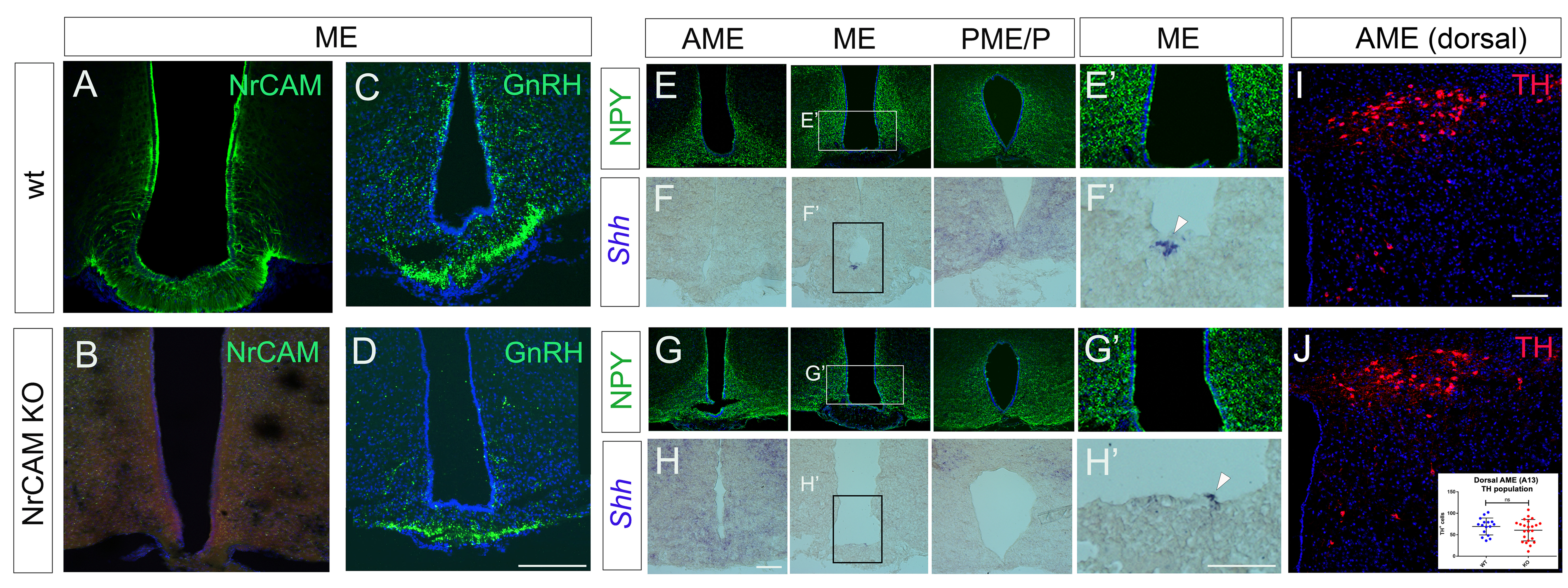

Supplement: Supplementary Figure 3 — Comparative analyses in wild-type and NrCAM KO mice. (A–J) Coronal sections through wild-type (A,C,E,F,I) or NrCAM KO (B,D,G,H,J) mice. Panels (A–D) show sections at level of ME, immunolabeled with anti-NrCAM Ab (n = 5 mice/genotype) or anti-GnRH Ab (n = 3 mice/genotype). Panels (E–H) show serial coronal sections through AME, ME and posterior regions of the hypothalamus, analyzed by immunohistochemistry to detect NPY or by in situ hybridization to detect Shh (n = 3 mice each/genotype). Panels (I,J) show sections at the level of the AME, showing the dorsal zona incerta (A13) TH + population (n = 3 mice/genotype). Inset in panel (J): Quantification of TH-positive A13 cells (same 3 pairs of wild-type and NrCAM KO adult mice as shown in Figures 3I,J,U,V). Each icon represents a single measurement. Bars show 1 SD either side of the mean value. Analysis by unpaired t-test showed no significant difference of TH+ cells in dorsal AME Zona Incerta (A13) population between wild type and NrCAM KO adult mice (p = 0.2543). Arrowheads in panels (F′,G′) point to Shh midline cells. Scale bars: 100 μm. [file Image_3.jpeg]

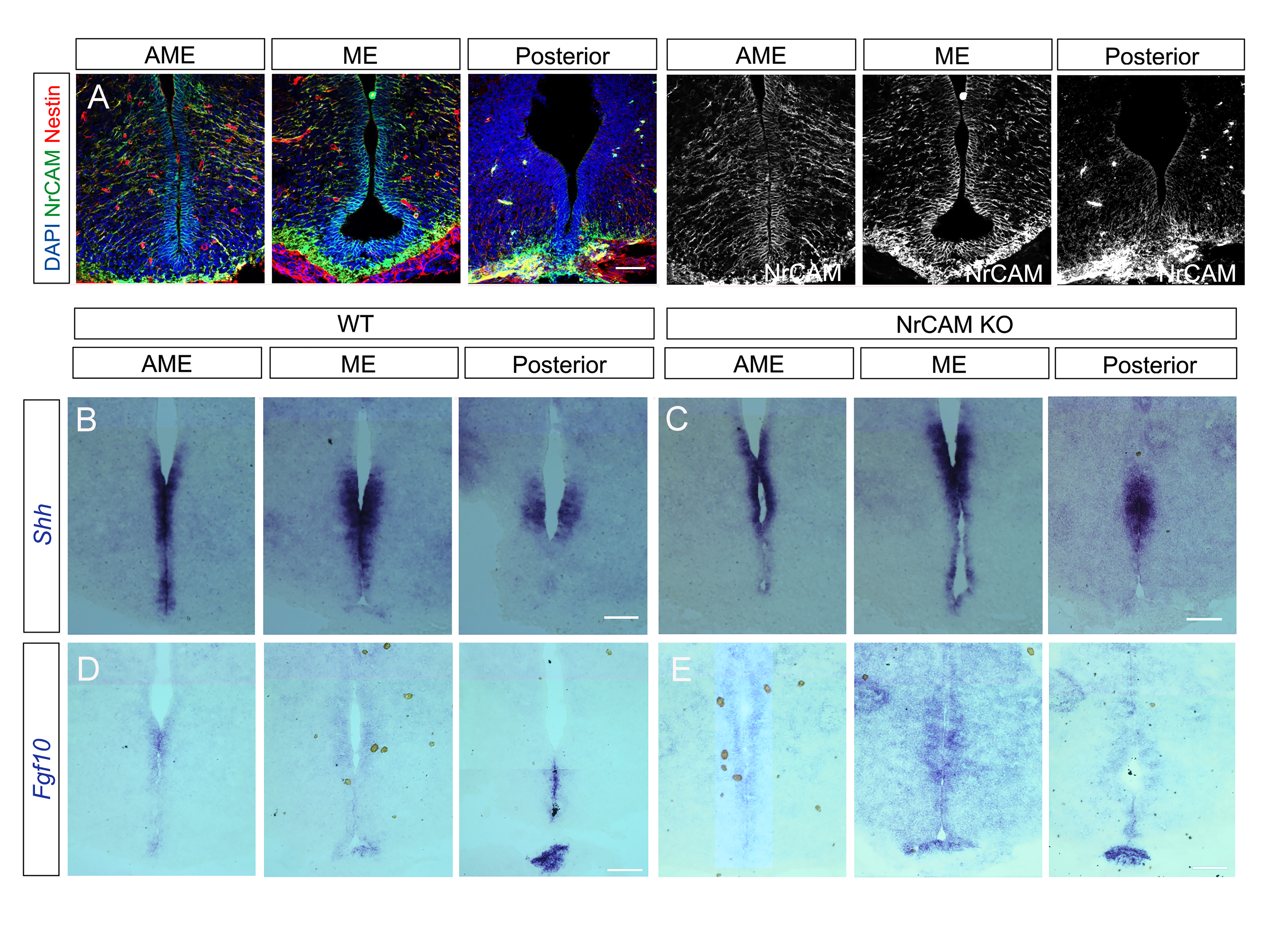

Supplement: Supplementary Figure 4 — Tanycyte progenitor markers at E16. (A–E) Representative serial coronal sections through AME, ME and posterior regions of the hypothalamus of a wild-type (A,B,D) or NrCAM KO mouse (C,E), analyzed by immunohistochemistry to detect NrCAM and Nestin (A) or by in situ hybridization to detect Shh and Fgf10. n = 3 mice/condition. Scale bars: 100 μm. [file Image_4.jpeg]

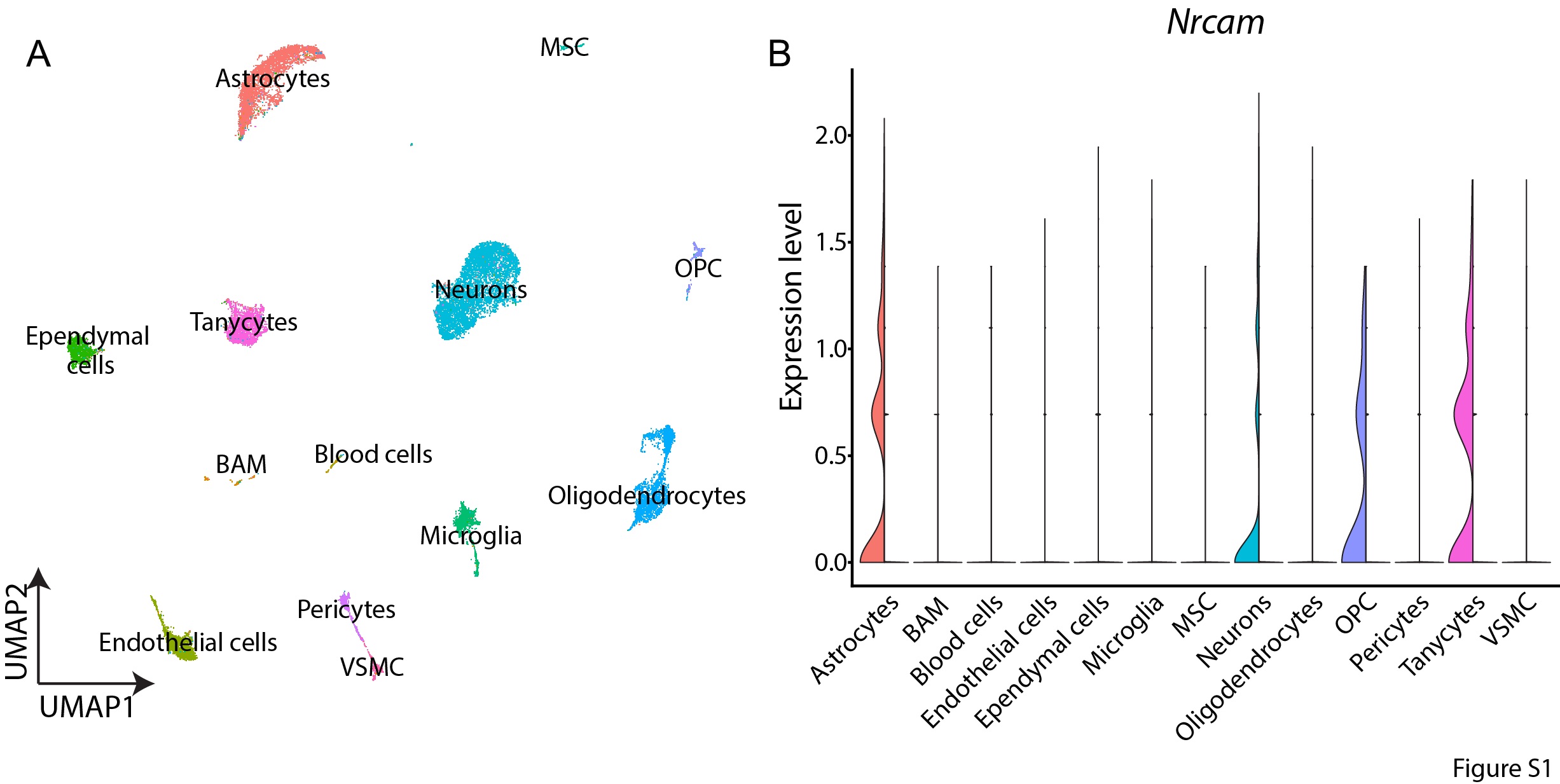

Supplement: Supplementary Figure 5 — scRNA seq analysis of the hypothalamus. (A) UMAP plots showing detected cluster. (B) Violin plots showing Nrcam expression across cell types. [file Image_5.jpeg]

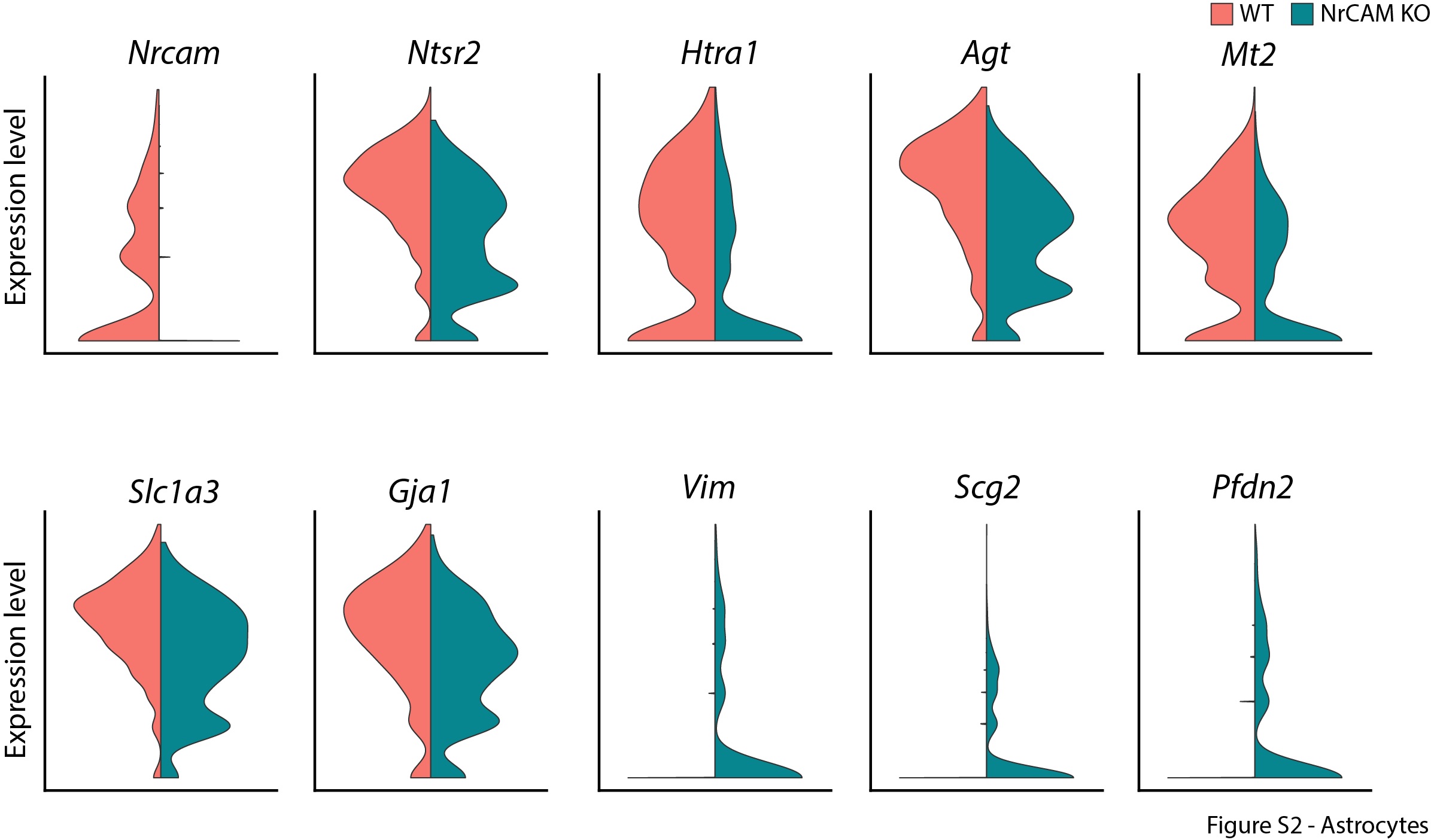

Supplement: Supplementary Figure 6 — scRNA seq of astrocytes from wild-type and NrCAM KO mice. Violin plots show differential gene expression of selective genes in astrocytes from NrCAM KO compared to wild-type hypothalamus. [file Image_6.jpeg]

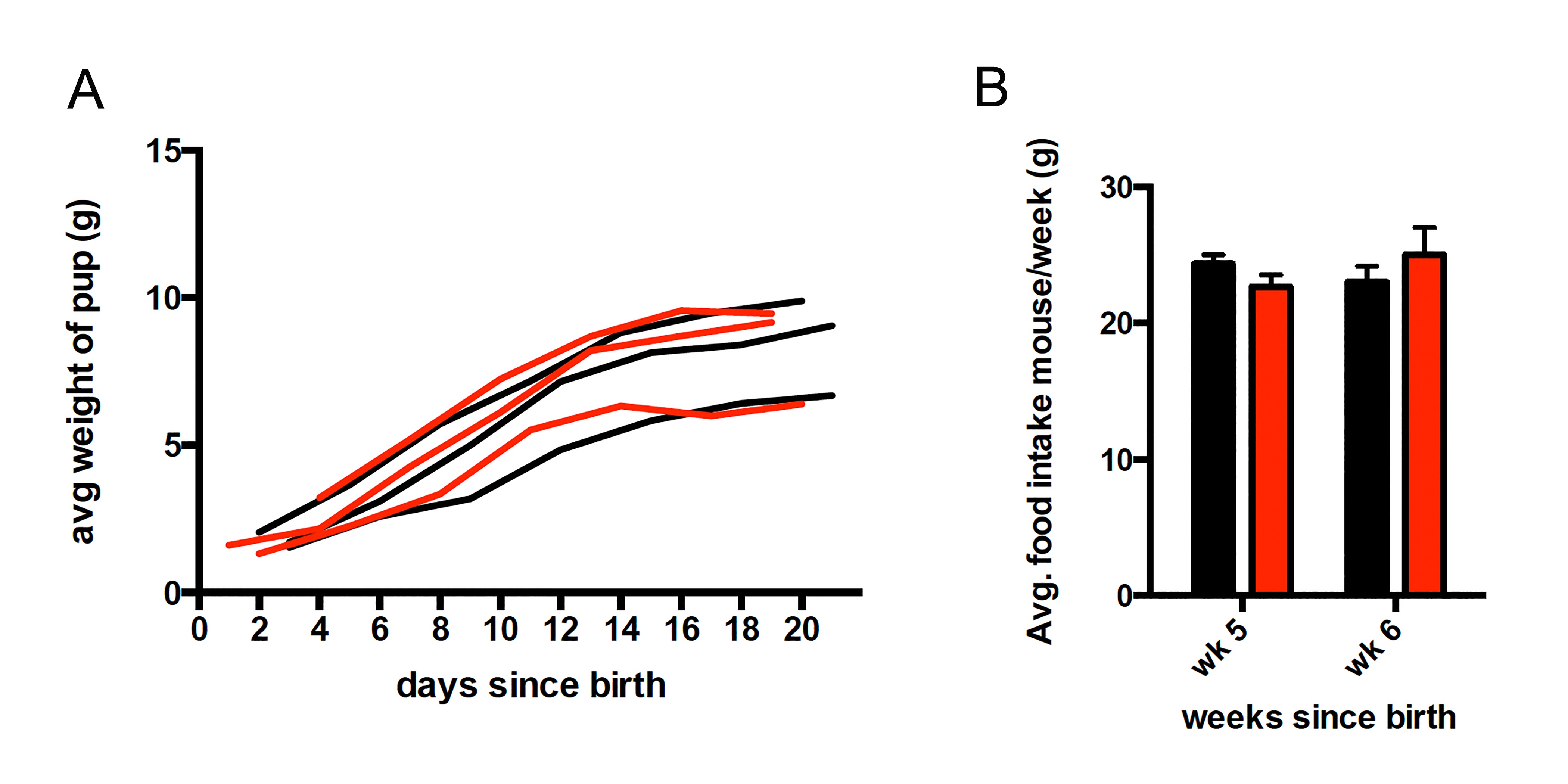

Supplement: Supplementary Figure 7 — Body weight and food intake in newborn-juvenile NrCAM KO and wild-type mice. (A) Body weight in 3 pups of each genotype; (B) average food intake of wild-type and NrCAM KO mice at 5 and 6 weeks (n = 6 per genotype). Values are mean ± SEM. Red—NrCAM KO; black—wild-type. [file Image_7.jpeg]
